# Estimating Cell Count and Distribution in Labeled Histological Samples Using Incremental Cell Search

**DOI:** 10.1155/2011/874702

**Published:** 2011-05-24

**Authors:** Oscar E. Meruvia-Pastor, Jung Soh, Eric J. Schmidt, Julia C. Boughner, Mei Xiao, Heather A. Jamniczky, Benedikt Hallgrímsson, Christoph W. Sensen

**Affiliations:** ^1^Department of Computer Science, Faculty of Science, Memorial University of Newfoundland, St John's, NL, Canada A1B 3X5; ^2^Department of Biochemistry and Molecular Biology, Visual Genomics Centre, Faculty of Medicine, University of Calgary, Calgary, AB, Canada T2N 4N1; ^3^Department of Cell Biology and Anatomy, Faculty of Medicine, University of Calgary, Calgary, AB, Canada T2N 4N1; ^4^Department of Anatomy and Cell Biology, College of Medicine, University of Saskatchewan, Saskatoon, SK, Canada S7N 5E5

## Abstract

Cell proliferation is critical to the outgrowth of biological structures including the face and limbs. This cellular process has traditionally been studied via sequential histological sampling of these tissues. The length and tedium of traditional sampling is a major impediment to analyzing the large datasets required to accurately model cellular processes. Computerized cell localization and quantification is critical for high-throughput morphometric analysis of developing embryonic tissues. We have developed the Incremental Cell Search (ICS), a novel software tool that expedites the analysis of relationships between morphological outgrowth and cell proliferation in embryonic tissues. Based on an estimated average cell size and stain color, ICS rapidly indicates the approximate location and amount of cells in histological images of labeled embryonic tissue and provides estimates of cell counts in regions with saturated fluorescence and blurred cell boundaries. This capacity opens the door to high-throughput 3D and 4D quantitative analyses of developmental patterns.

## 1. Introduction

In many areas of biomedical research including clinical pathology, cell counts obtained from images are crucial data for diagnosing patients or for addressing hypotheses about developmental or pathological processes. Manual cell counting is challenging in that it typically requires a specialist such as a biologist or a pathologist to identify and characterize different cell types. Even then, manual cell counts are subjective. To date, several stereological tissue analysis methods have been developed with the aim of accurately estimating cell counts in a given tissue [1, 2]. However, traditional stereological techniques require elaborate production and sampling of representative tissue samples, which is both time consuming and labor intensive.

Recent technological advances in microscopy now enable high-throughput imaging of thousands of cells in a short time [3–5]. Further, using high-throughput slide scanners allows the rapid collection of high-resolution data for serial sections [6]. These serial sections can then be reconstructed virtually in 3D. The 3D reconstructions can then be statistically analyzed using morphometry to quantify variation among samples. Manual cell counting would be a tedious and time-consuming approach for processing such large datasets and may even be subject to inaccuracies due to human error, as noted by several authors [4, 7–10]. In the context of the technological developments previously mentioned, computer-automated cell identification and counting could accelerate data collection. Importantly, this would greatly facilitate high-throughput histomorphometry, permitting large-scale studies of cellular processes that would not be feasible if done only by a manual process. 

A common challenge for computerized cell counting methods arises from the great variation in the morphology and size of cells [8], compounded by the availability of many different histological, staining, and imaging techniques. This led to the development of a variety of computer-automated cell counting tools and the elaboration of sophisticated methods such as the one described by Carpenter et al. [4, 11], where a pipeline of image-processing techniques can be defined and then applied to a series of images. Often, the solutions for cell counting are based on segmentation methods applied after edge detection [3, 10–15]. These methods can handle cells of various sizes and shapes, since cells are primarily identified by the detection of intercellular boundaries and the separation from the background by edge detection. 

**Figure 1:**
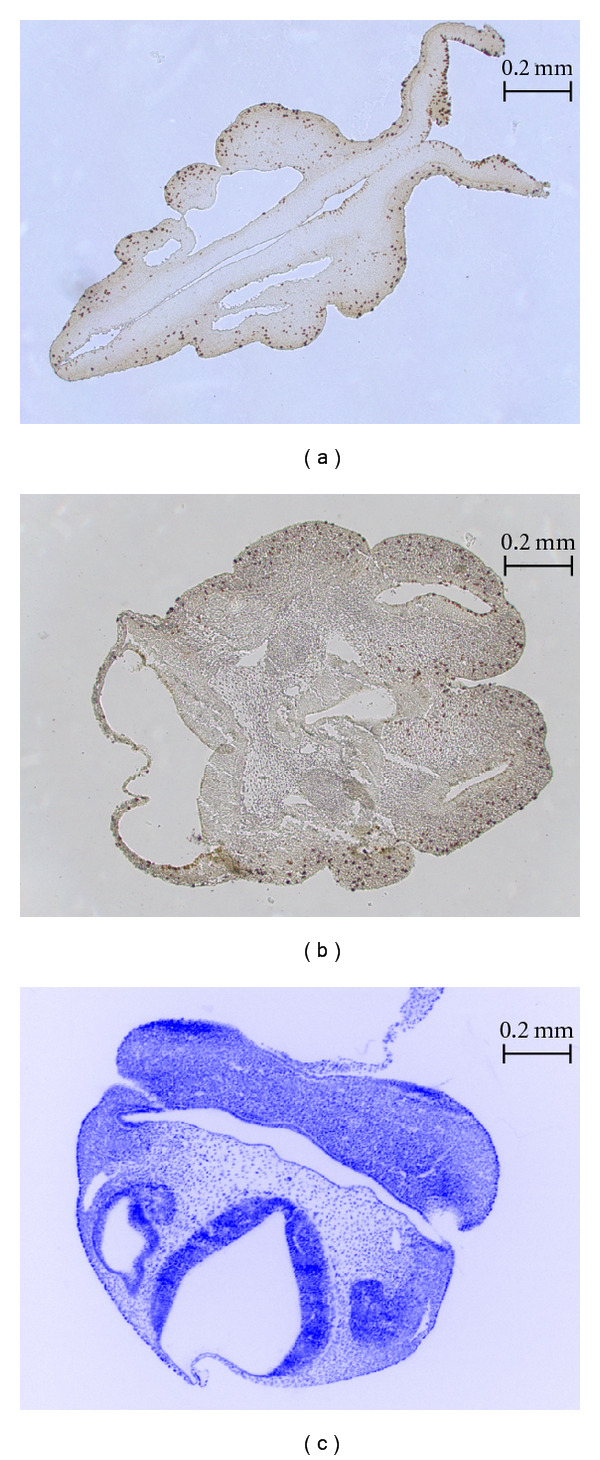
Mouse embryo head tissue samples considered in our study. (a) and (b) show dark-stained nuclei of proliferating cells, whereas (c) shows nuclei of proliferating cells stained to fluoresce under UV light (background inverted to white for viewing clarity).

Images of developing embryonic tissue, which is of particular interest to developmental biologists, can be difficult to process using edge detection methods, particularly when the density of the cells in the tissue is high and blurring, or if the boundaries between cells disappear entirely. An example of this occurs in fluorescence microscopy, when excess saturation in the brightness signal seeps into a cell from adjacent cells labeled with fluorescent dye. The blue-saturated regions of the sample shown in Figure 1(c) illustrate this case.

An alternative approach to obtain cell count estimates that are not based on edge detection is texture analysis, where image segmentation is followed by cell quantification using a measure of the average cell size [16, 17]. Malpica et al. present a method for quantification of cells in images of cell cultures that consists of first classifying regions of the input image into different classes, and then estimating the total number of cells simply by dividing the total surface of the classified region over the average surface covered by any cell [17]. However, these methods would be clearly insufficient to support morphometrics studies. To support developmental morphometric analysis, these methods would need to provide the approximate locations of the cells within the samples and not just estimates of total cell counts. This location information is necessary in some applications where the actual distribution pattern of cells in the tissue is biologically relevant. For example, in our morphometrics application, the information about the location of the cells is required to model cell distribution in 3D space and assess the impact of the proliferating cell locations on the development of the cleft lip and cleft palate facial morphology disorders. 

We have developed software for cell identification and localization in images depicting the roughly spherical proliferating cells commonly present in embryonic tissue. Figure 1 shows how, through the application of different staining methods, embryonic cells become clearly differentiable from the background and from other cells by having a particular color under brightfield microscopy or by fluorescing under UV light. Our approach identifies probable cell locations based on color information and average cell size. Our method is derived from image-processing techniques that produce Poisson-Disk distributions commonly used for image-based sampling and nonphotorealistic rendering [18, 19]. The method proposed is simple to implement and requires little information from the user. The primary identification criteria are the color range of the cells and the average cell diameter. 

We have compared our software with the Image-Based Tool for Counting Nuclei (ITCN) [12, 15], a cell counting solution available as a Java plug in for ImageJ. ITCN is an edge detection-based method that computes the Laplacian of Gaussian (LoG) to identify nuclei [13]. Users of ITCN must enter an estimated cell width (*sic*) and an estimate of the minimum distance between the nuclei, a value set by default at half the width. From this information, ITCN provides total cell counts for an image or a region of interest and an output image that shows the cell locations.

## 2. Methods

### 2.1. Stippling, Cell Counting, and the Poisson-Disk Distribution

Our cell identification algorithm is inspired by Mould's progressive stippling [19], a technique where stipples are placed on an image using a graph extracted from the input image and Dijkstra's shortest path search algorithm [20, 21].

Many graphics applications rely on the production of Poisson-Disk (PD) distributions [18], and stippling techniques are commonly used in nonphotorealistic rendering to represent, in an aesthetically pleasing way, a grayscale image while preserving the characteristics of a PD distribution as much as possible [19, 22, 23]. PD point distributions are defined as sets of regularly distributed points or particles where all points are separated from each other by a certain minimum distance, called the radius of the distribution. Since the cells in our study are roughly round in shape and tend to have a regular size, we draw a parallel between cells in histology samples and points that follow the PD distribution. As our algorithm progresses, the Poisson-Disk distribution, which is typically used to evenly disperse points on a plane, becomes apparent only in those areas of the image where the colors of the cells match the user-defined color range. ICS uses the average cell diameter to place markers for cell locations, so the radius of the PD distribution in those areas is half the average cell diameter. The Poisson-disk distribution cannot simply be substituted by a random uniform distribution, because it lacks a distribution radius, and in the absence of an average cell diameter, there are no criteria which can be used to determine how many cell location markers (dots) should be placed in regions covered by cells.

### 2.2. Overview of Incremental Cell Search

**Figure 2:**
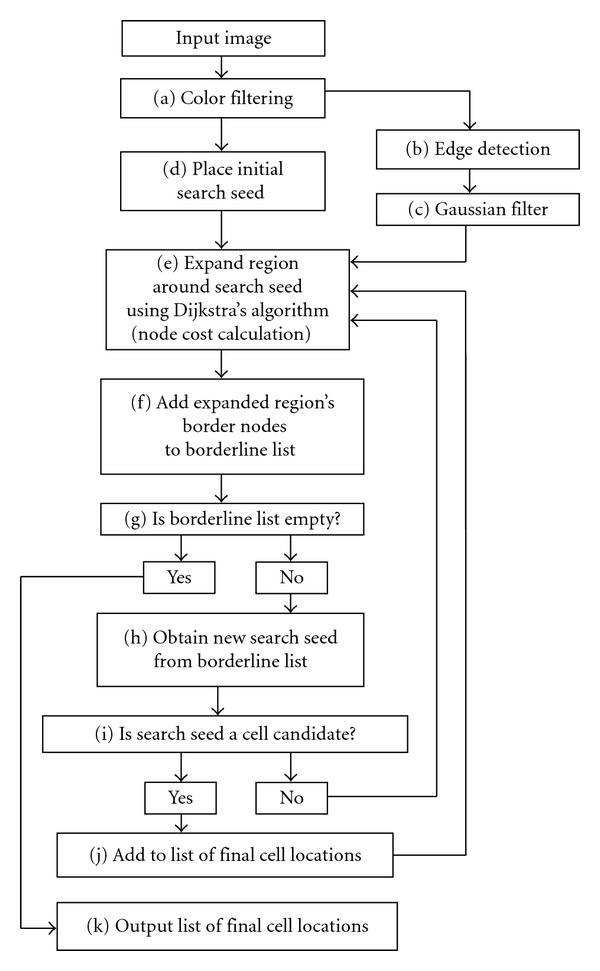
ICS overview flowchart.

**Figure 3:**
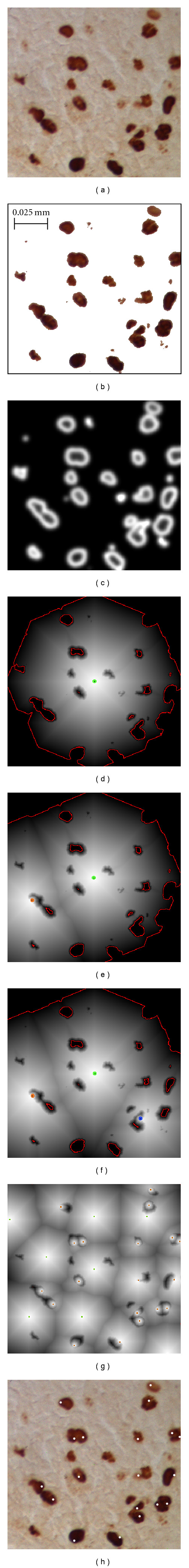
Step-by-step overview of ICS. (a) A slice sample using DAB staining under brightfield microscopy. (b) The original image after color filtering. (c) The diffuse edge detection buffer. (d) Expansion region after the first search iteration, with the initial seed placed at the center of the image (the green dot). The red outline in (d) (the border of the expansion region) illustrates the edges from where future seed points will be chosen. Figures (e) and (f) show the expansion regions after the second and third iterations, respectively. The new seed shown in (e) (the orange dot) was located on the red outline in (d), and the new seed shown in (f) (the blue dot) was located on the red outline in (e). (g) shows the result after all the expansions have taken place. (h) shows the positive cell location markers placed on the image once the algorithm ends; where two markers appeared in close proximity, the algorithm has detected two adjacent cells.

To find cells using ICS, the user indicates the color or grayscale intensity of the target cells (resp.), a margin of tolerance that defines the amount of acceptable variation with respect to the base color, and an average cell diameter.  Figure 2 provides an overview in the form of a flowchart of the process undertaken by the software, while Figure 3 illustrates some steps of ICS as it goes through the sample input image shown in Figure 3(a). The first step is color filtering (Figures 2(a) and 3(b)), where the image is segmented in two regions according to the user-provided color range: the regions where stained cells are found and a blank background elsewhere. Next, we obtain an auxiliary image for later use: an edge-detection filter is applied on the original image (Figure 2(b)) and then passed through a Gaussian filter (Figure 2(c)). We call it the “diffuse edge-detection buffer” as shown in Figure 3(c). Subsequently, we place a seed at the center of the image (Figure 2(d)) and green dot (Figure 3(d)) and use a graph analogy to apply Dijkstras's shortest path algorithm (Figure 2(e)), starting from this seed to create a region around it that grows until the average cell diameter is exceeded. Once Disjkstra's algorithm has been applied (Figure 3(d)), we insert all the elements (pixels) on the border of the expanded region in a First-In First-Out (FIFO) list called the “Borderline list” (Figure 2(f)), shown as a red border between white and black regions in Figure 3(d). Note that each red pixel represents one element from the list of borderline nodes. Together, the elements in this list form red contours or borders enclosing the regions of expansion covered by Dijkstra's algorithm. If this borderline list is not empty (Figure 2(g)), we remove the first element of the borderline that exceeds the average cell diameter and make it a new search seed (Figure 2(h), orange dot in Figure 2(e)), then check whether it falls within the color-filtered region where cells are found (Figure 2(i)). If it does fall within this region, we consider it a valid cell location marker and add it to the list of final cell locations (Figure 2(j)). If it does not, we simply start the next iteration of Dijkstra's shortest path search and expand the region around it (Figure 2(e)). This process is repeated constantly, adding seeds and updating the list of final cell locations, the regions of expansion (white regions in Figures 3(d), 3(e) and 3(f)), and the borderline list (red edges in Figures 3(d), 3(e) and 3(f)) until the borderline list is empty, case in which we output the list of final cell locations, (Figure 2(k)). Figures 3(e) and 3(f) show the expansion regions after the second and third iterations.  Figure 3(g) shows the result after all the expansions have taken place, and Figure 3(h) shows the positive cell location markers placed on the image once the algorithm ends. The process of iteratively adding seeds regardless of whether they identify cell locations or not ensures that the algorithm eventually covers the whole image when searching for cell locations within the image. More details on the individual steps of this process and the rationale behind it are found in the following sections.

### 2.3. Color Filtering

After user input is complete, ICS first runs the input image through color filtering (Figure 2(a)). Color filtering removes all areas that do not match the user-specified color range (Figure 3(b)). The user needs to indicate the base color of the stain in the sample because stains can come in many different colors. The amount of variation in the color of the stain depends on the type of stain used, on how the stain is fixed on the specimen, and on the type of light used when photographing the sample. Only the user can assess what is an acceptable amount of color variation. For this reason, the margin of tolerance is determined by the user rather than computed by a formula, and it is important that the user carefully selects a representative color and defines an adequate threshold (or margin of tolerance) for the valid color range of cells in the image, according to the staining and the illumination methods used. The margin of tolerance is a single scalar value that limits the range of colors that are considered similar to the base color considering all three channels. 

To help the user visualize the selected region resulting from the user-provided base color and margin of tolerance values, the user can run ICS's graphical interface to obtain a color-filtered version of the input image before execution of the whole algorithm. Alternatively, the user can run the freely available GNU Image Manipulation Program (GIMP) [24]. GIMP's color picker and color-based selection tools allow the user to interactively select the regions that define valid cell targets to produce a color-filtered version of the input. The use of GIMP is specifically recommended as an alternative for determining the input parameters to our tool (base color, range of variation, and average cell diameter), not as an alternative to our tool in its own. GIMP is proposed when the user prefers to interactively experiment with the color filtering parameters, by using an interactive sliding bar to regulate the color tolerance margin and an interactive line-measuring tool (ruler) that can be used to determine the average cell diameter in pixels, prior to using our tool with the preferred parameters. 

Regardless of whether ICS or GIMP is used to select the values of the base color and its margin of tolerance, the user does not need to compute the margin of tolerance that defines the amount of acceptable variation with respect to the base color for each color channel. ICS calculates whether the color of each pixel in the image falls within the user-provided margin of tolerance by computing the Euclidean distance between the base color's RGB triplet and each pixel's own RGB triplet. If (and only if) this distance is less than the margin of tolerance, the color is considered to be within the acceptable range of variation. 

### 2.4. Placement of the Initial Search Seed

After color filtering, and once the diffuse edge detection buffer has been obtained (see Section 2.7), a search seed is automatically placed by the system at the center of the image (Figure 2(d), green dot in Figure 3(d)). The initial seed is just the starting point for exploring the image, and the user does not need to specify a seed location at any time when using the system. In fact, the seed could have been placed randomly somewhere on the image. Its location is rather irrelevant when determining total counts because the algorithm produces a Poisson-disk distribution, regardless of the initial cell location. The exact distribution of cell location markers will change if the initial seed is placed at a different place each time the process is executed. However, the regions for placing cell location markers depend on the region defined by the color filter and not on the initial seed location. The general trend is that counting results will be more robust when the number of cells present in the sample is high.  Table 3 (presented in more details in Section 3) shows that when counts are low (average 26.8 cells per sample), the coefficient of variance (CV) is 8.9% and, as the number of cells in the sample increases (average of 374 cells per sample), there is less variation in the results (2.7% CV).

### 2.5. Region Expansion Using Dijkstra's Algorithm

The initial seed is used as the starting point for an expansion operation performed using Dijkstra's shortest path search algorithm [20, 21] (Figure 2(e)). For illustration purposes, we use a graph analogy, where each pixel in the image represents a node in a graph, and the edges of the graph represent the connections between one pixel and its neighbors in the image. Unless a pixel is located at the border of the image, it is connected to 8 neighbors. The node costs needed for Dijkstra's search algorithm are computed based on the distance from the seed, the color or tone intensities of the pixels along the path, and the edge intensity of the pixels. To guide the search for cells and to store the traversal information as the algorithm progresses through the image space, our implementation uses several image buffers shown in Figures 3(b)–3(f).

### 2.6. Computing the Node Cost during Region Expansion

An essential feature of Dijkstra's shortest-path search is that every edge in the graph has an associated cost that contributes to the total cost of a given path. In ICS, the cost for traveling from one node to the next (i.e., from one pixel to the next) is the sum of the cost stored in the source node and the computed cost of the target node. Three elements contribute to the cost of a target node: the distance to the seed from the target, whether the color (or the grayscale tone) of the underlying pixel matches the target cell color (within the user-provided range), and the intensity of the gradient at the location of the node.

First, we calculate the base cost of a target node as the distance between the source pixel (*x_s_, y_s_*) and the neighboring target pixel (*x_t_, y_t_*),

(1) $$\;\text{Base}\;\text{Cost}\;=\sqrt{{\left(x_{s}-x_{t}\right)}^{2}+{\left(y_{s}-y_{t}\right)}^{2}}.$$

The base cost equals 1 for two horizontally or vertically aligned pixels and equals $\sqrt{2}$ for diagonally adjacent pixels. Next, we test whether the pixel color at the visited node falls within the user-indicated color range. If the pixel falls outside the target color range, the node cost is reduced to have a larger expansion region,

(2) Node  Cost  =  Base  Cost×0.10.

Otherwise, we increase the base cost by an amount proportional to the value of the gradient intensity at the underlying pixel,

(3) $$\begin{array}{cc} & \text{Node}\;\text{Cost}\\ & =\text{Base}\;\text{Cost}\times \left(1+\text{Gradient}\;\text{Intensity}\times \text{Edge}\;\text{Weight}\;\text{Factor}\right). \end{array}$$

The gradient intensity is a value between 0 and 1 that indicates the strength of the edge detection buffer at the corresponding pixel location (see “Diffused Edge Detection”). The Edge Weight Factor (EWF) is a user-adjustable value between 0 and 1 (default value of 0.5). Equation (3) can be used when the average cell diameter is close to 10 pixels wide. As image resolution increases, the average cell diameter increases as well. To obtain consistent counts at increased image resolutions, we use the following formula to compute the node cost:

(4) $$\begin{array}{cc} & \text{Node}\;\text{Cost}\\ & \;\;=\text{Base}\;\text{Cost}\times (1+\text{Gradient}\;\text{Intensity}\;\\ & \;\;\;\;\;\;\;\;\;\times \frac{\left(\text{Average}\;\text{Cell}\;\text{Diameter}+8\right)}{18}\\ & \;\;\;\;\;\;\;\;\;\times \;\;\text{Edge}\;\text{Weight}\;\text{Factor}). \end{array}$$

This formula takes into consideration the average cell diameter (a user-provided value) in the input image. The constant values and the linear form of (4) were obtained experimentally and ensure that the counting results are consistent across multiple resolutions for a wide range of cell diameters, from 6.5 to 42 pixels (a ~32-fold variation in cell surface coverage, as shown in Table 3).

### 2.7. Diffused Edge Detection Buffer

To obtain values for gradient intensity of the input image, we apply an edge detection filter to it and store the resulting image in the edge detection buffer (Figure 2(b)). Edge detection is an operation where results are dependent on the resolution of the image or the magnification of the specimen. To compensate for this dependency, we apply a Gaussian filter that extends the edge-detected region as a function of the user-provided cell diameter value (Figures 2 and 3(c)). The kernel size of this filter is 50% of the cell radius (0.25 × input cell diameter). This filter produces a diffuse band around the edge of the color-filtered regions, which is used in conjunction with the EWF and the cell diameter value as shown in (3). Since the blurring step reduces the energy of the individual pixels, we enhance the contrast of the edge detection buffer by stretching the histogram after Gaussian filtering using ImageJ's contrast enhancer function. This function automatically sets the upper and lower histogram levels given an (default) input value of 0.5% saturated pixels. As a result of this stretching, pixels in this buffer reach the maximum edge-intensity value of one on the edges, and the intensity values decrease towards zero in the transition to regions with no edges.

The EWF introduced in (3) can be used to regulate the amount of cell candidates in locations close to the edges of cell clusters and in the detection of isolated cells. To increase the cost of the traversal and to detect cells in border regions of isolated cells and cell clusters, the EWF should be adjusted to a value that matches the relevance of the edges on an image. The recommended values are 0.5 (default) to 1. An EWF value of 0.5 contributes half of the traversal cost needed to reach the center of a cell that lies on the border of the color-filtered region. This facilitates the detection of cells that lie on the boundaries of the color-filtered region. This value can be increased towards 1. This would produce an increase in the presence of cells at the edges of the color-filtered regions.

### 2.8. Iterative Region Expansion

The process of expansion starts from the location of the search seed and stops when the cost to visit a node exceeds a certain threshold (Figure 2(e)). The threshold is the user-provided value for the average diameter of the cells in the image. Figure 3(d) illustrates the expansion after the first iteration. When the expansion stops, we add the pixels that lie on the border of the expansion region to a list of potential starting points for future searches (Figure 2(f)). This list is shown forming a red borderline in Figures 3(d)–3(f). Provided that the list of borderline seeds is not empty, we retrieve an element from this FIFO list and make it a candidate for the becoming new search seed for the next iteration (Figure 2(h)). Before we start an expansion, we check whether the candidate's current cost still exceeds the threshold, the case in which we consider it a search seed. If the candidate cost does not exceed the threshold, we discard it and retrieve another candidate from the list of borderline elements. If the search seed's color is within the color range specified by the user (Figure 2(j)), the seed is tagged as the location of a new cell (such as the orange dot shown in Figure 3(e) and subsequently the blue dot in Figure 3(f)), then added to the list of positive cell locations, and used to start the search for the next seed. If the search seed's color is outside the color range specified by the user (such as the green dot in Figure 3(d)), the seed cannot be considered a potential cell location, so we do not add it to the list of cell locations, but we still use it to start a new search since we still need to cover the whole image in search of potential cell locations and to discard irrelevant regions. 

**Figure 4:**
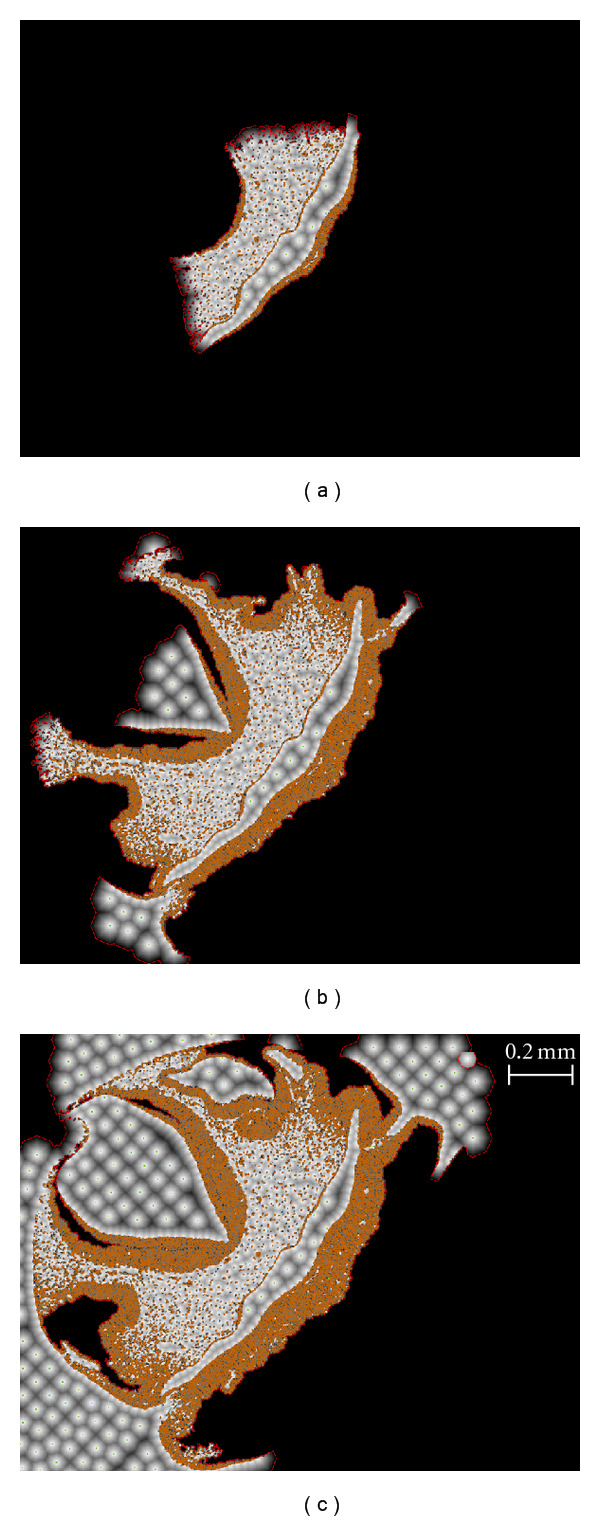
ICS progress. The 3 frames above show how ICS searches through an image over time. The algorithm starts from the center of the image where the first search seed was placed (first frame). For each frame, orange regions (more visible in the second and third frames) represent regions with positive cell locations, and white regions represent regions where no cells were found. The regions covered by the algorithm are enclosed by a thin red boundary, and the dark regions have not yet been explored by the algorithm.

We run iterations of this search procedure that progressively covers the image (see Figure 4) until there are no more seed candidates in the borderline list, which occurs when the image has been completely explored (Figures 2(k) and 3(g)). Figures 3(e) and 3(f) illustrate the second and third expansions, respectively. The process of iteratively adding seeds ensures that the algorithm eventually covers the whole image, regardless of the location of cells within the image.  Figure 11 illustrates how the proposed method can contribute to deal with challenging regions where images are overexposed to the staining material, and where existing methods perform poorly. For instance, Figure 11(f) shows a Poisson-disk distribution of cell markers in the saturated intensity (white) regions and how ICS uses the average cell diameter to place markers for cell locations. Overall, our results suggest that our method provides higher precision for large number of counts and the ability to process images at different resolutions for a wide range of cell sizes (see Table 3). After the iterations are completed, the final step is the display of the list of positive cell locations found (the white dots in Figure 3(h)), which can optionally be stored in a text file.

### 2.9. Criteria for Cell Identification

In our method, two main factors influence the identification of a potential cell. The first is whether the color value at the potential location matches the user-provided color range. The second is whether, in a region where the pixels match the target color value, a cost threshold has been exceeded while sweeping the image with Dijkstra's algorithm. 

For the cost threshold, we use the average cell diameter parameter to discard small traces of cells and prevent overcounting. In some cases, such as in Figure 3(h), it may seem that some cells are missed. However, counting all traces of cells in an image will inevitably lead to overcounting, because not all stained flecks in an image correspond to complete cells. The reason for this is that the specimen is cut into thin slices. Small flecks of stain in one slice are likely to originate (bleed) from cells in adjacent slices. For instance, in Figure 3(h), some small stained regions around the center of the image were not counted as cells based on their small size; those will be counted in the image of the next slice, once they are shown in full size.

**Figure 5:**
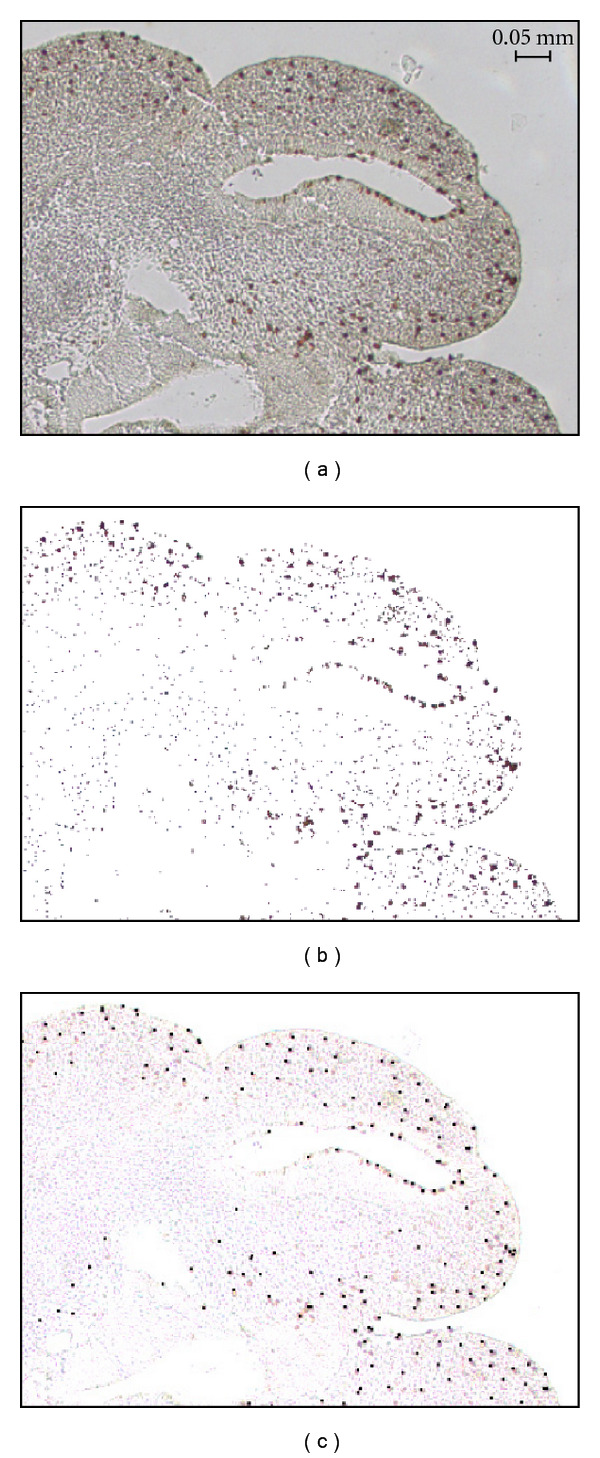
The use of color filtering for estimating cell locations. (a) Details of a portion of the original tissue sample shown in Figure 1(b). (b) The same region after color filtering. (c) The cell locations shown as black dots.

Figure 5 illustrates filtering by color, where a significant amount of “noise” in the form of irrelevant features is conveniently removed from the image. The original image (Figure 5(a)) shows part of a slice with a particular type of nuclei stained with a dark purple dye. ICS ignores the regions that fall outside the target color range (Figure 5(b)) and estimates cell locations exclusively within the regions that match the target color (Figure 5(c)). This approach is very effective at removing noise from stained samples, a common source of error for many automated counting methods. 

**Figure 6:**
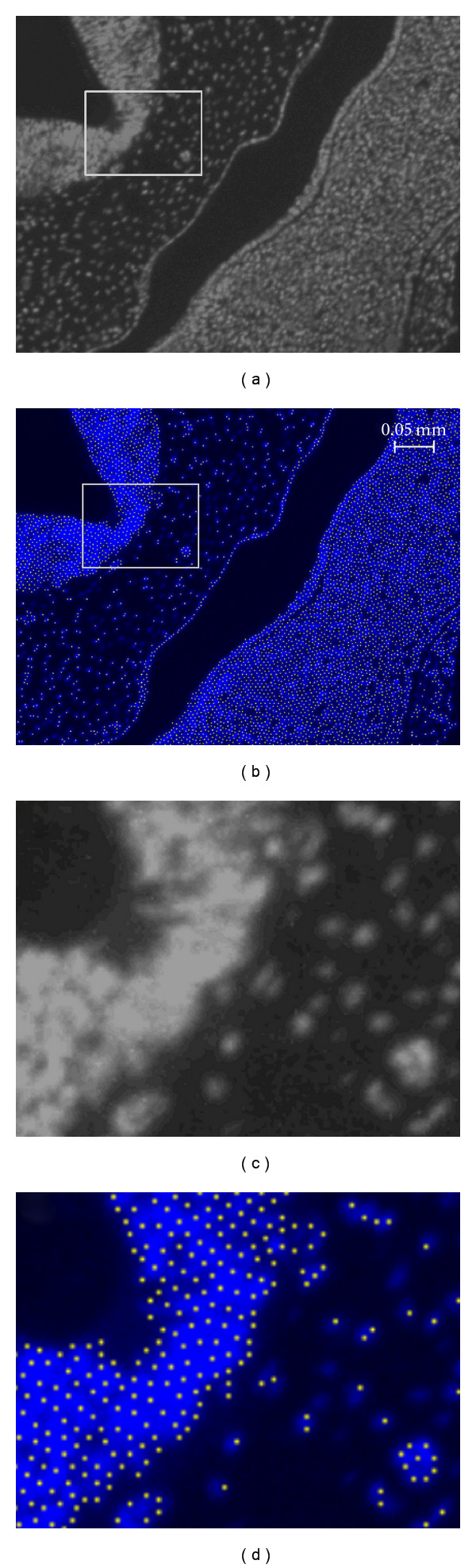
Details of ICS results. (a) Detail view of a DAPI-stained sample obtained through brightfield microscopy. The image has been converted to grayscale and contrast enhanced for viewing clarity. The blue regions in (b) show the color of the original image and correspond to the white regions in (a). Figures (c) and (d) are zoomed windows (4x magnification) of (a) and (b), respectively. The yellow dots in (d) show the cell location markers placed by ICS in (c).

As described previously in the overview, the iterative expansions that take place when looking for potential seed locations finish when the traversal cost threshold is exceeded. When an expansion stops, all nodes in the expanded region's border have costs above the threshold. This implies that elements removed from the borderline list are expected to exceed the user-defined intercellular distance with respect to the original expansion seed. Therefore, those elements become valid candidates as cell location markers. When an element taken from the borderline list falls within the user-defined color range and its cost still remains above the threshold, we consider it a new cell location and add it to the final list of cell locations. Note that search seeds inserted in the final list of cell locations can be located anywhere within the cell and do not indicate the center of the cell, as shown in Figure 3(h).  Figure 6 shows the placement of cell marker locations on a section sample obtained through fluorescent lighting. 

### 2.10. Software Availability

The software is implemented in Java and freely available for download from http://www.visualgenomics.ca/~omeruvia/research/ics.html. To use the program, the user needs to select an image, fill in the values in the input dialog describing the average cell diameter and the cells base color and range, and the Edge Weight Factor, and then press the Start Counting button. In addition, users can select the “save cell locations file” checkbox to save the cell location coordinates in a text file or show the auxiliary image buffers. This implementation makes use of functionalities available through the ImageJ libraries. The ImageJ distribution is available at http://rsbweb.nih.gov/ij/. Additionally, our implementation uses functionalities available through the Java 3D vecmath library from Sun. The Java 3D distribution is available at http://java.sun.com/javase/technologies/desktop/java3d/.

### 2.11. Validation and Statistical Analysis

**Figure 7:**
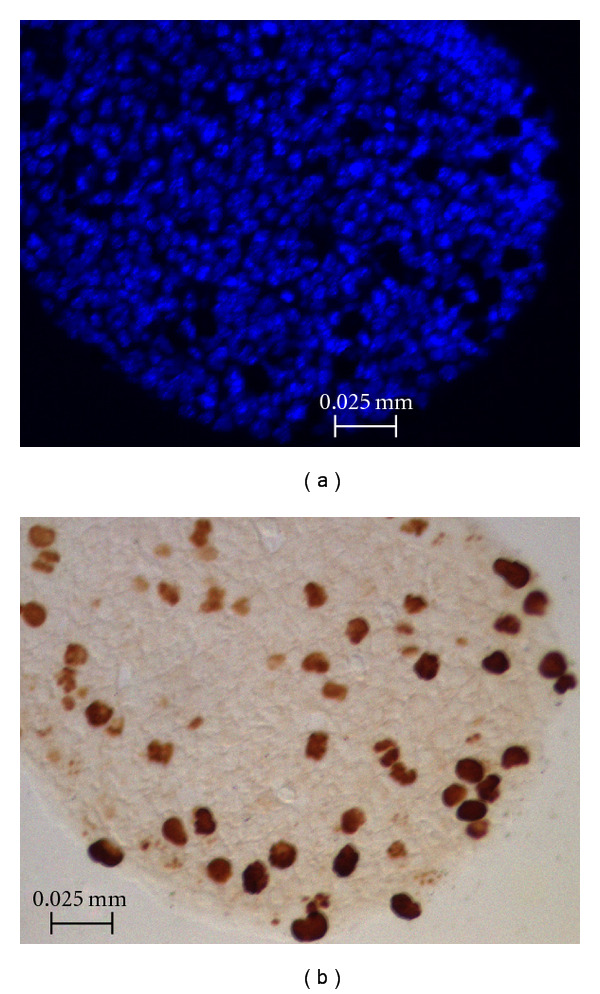
Sample tissue slices used in the experiments. (a) DAPI-stained nuclei shown under a UV light source. (b) DAB-stained cells shown under a bright-field light source.

We validated the ICS method using 21 serial sections from a mouse embryo head imaged under two illumination conditions for a total of 42 images. Specimens were embedded in paraffin, sectioned at 10 microns, and stained using a DNA-binding fluorescent stain, the 4′,6-diamidino-2-phenylindole (DAPI). DAPI stain makes nuclei appear as bright blue spots under UV light (Figure 7(a)). Proliferating cells undergoing mitotic division (M-phase) were labeled using anti-P-H3 rabbit polyclonal IgG, Upstate Biotechnology (Lake Placid, NY), a standard immunolabel known as DAB, that recognizes histone protein modifications specific to mitotic chromosomes of dividing cells. Labeled cells were next tagged with DAB staining and appear as dark brown spots under brightfield microscopy (see Figure 7(b)) [25]. Each embryo section was digitally photographed with a Zeiss Axioplan 2, equipped with a Zeiss AttoArc 2 HBO 100 W lighting apparatus, at magnification 200x in order to capture a clearly defined subset of the cells on the slice. These images are in the range of 322 to 1093 pixels wide and 251 to 854 pixels high and are provided in the software distribution of ICS. In these images, the number of DAPI-stained (fluorescing) nuclei equals the background number of cells present in a section, while the number of DAB-stained (dark brown) nuclei equals the number of proliferating cells present in the same section. In addition, DAPI images were autocontrasted using ImageJ (Image→Adjust→Brightness/Contrast→Auto) to reduce variation in the brightness of the images. 

We compared the counts obtained using ICS and ITCN [12, 15] against the manual counts done independently by two trained human observers. ITCN is a tool for counting nuclei that is publicly available as a Java plugin to ImageJ, and commonly used as a reference for cell counting [10, 13, 26–29]. ICS counts were all produced using the default EWF value of 0.5. To adjust for variation of the brightness of the blue signal (RGB color triplet 〈0,0, 255〉) in the set of DAPI-stained nuclei images, we selected one of two levels of color tolerance (120 and 135). We selected these values by visually assessing the coverage of the regions shown by the color filter preview tool provided by the software. We found that these two levels adequately covered the regions of interest in our experimental images. Additional processing steps to obtain the ITCN counts were the conversion of images to grayscale, the grayscale inversion, and finally the autocontrasting of the DAPI-stained (fluorescent) images. This was necessary since ITCN only processes grayscale images, and it only detects dark peaks.

The statistical validation aimed to quantify both measurement bias and measurement error for cell counts on the DAB (proliferating cells only) and DAPI (all cells) sections, as well as for cell proliferation. Cell proliferation is the ratio of DAB-positive cells to the total number of DAPI-stained cell nuclei. We assume the average manual count to be the gold standard for comparison. Since each individual counter made a separate count of each section, we average the values produced by these two individual counters to obtain the average manual count. Bias is then quantified as the average difference between the cell counts obtained using the computer-based methods and the average manual count. ANOVA was used to determine the statistical significance of bias. Measurement error is quantified as the variation between measurements of the cell counts of the two automated methods with respect to the average manual count. To quantify measurement error for the two methods, we obtained the coefficient of variation (CV) for the deviations from the average manual count for each method. We used the CV, which is defined as the ratio of the standard deviation to the mean, rather than the variance, since the CV is a normalized measure of variation which allows direct comparison of the three variables examined. Statistical comparisons of the variances about the mean deviations for each method were based on Levene's test, which is robust to deviations from normality [30]. To assess the linearity of the automated counts with respect to the manual counts, a correlation analysis was performed. In addition, Bland Altman plots were generated to assess the difference between both ICS and ITCN's counts against the average of manual and automated counts. 

The performance of ICS for handling images at multiple resolutions was tested using the same dataset previously described. To produce copies of this set at multiple resolutions, the original set of images was digitally resized. Four additional sets of images were generated using this procedure, corresponding to magnifications 400x, 100x, 50x, and 12.5x each. The base value for the average cell diameter was 23 pixels, so the method was tested against the corresponding average cell diameter values of 34.4, 16.2, 11.5, and 5.75 pixels. The changes in the resolution of the additional sets correspond to a 100% increase (400x) and a decrease to 50%, 25%, and 6.25% of the surface covered by a cell from the original dataset. Unlike our method, in the case of ITCN, it was not possible to obtain consistent counts at multiple resolutions by only changing the value of the input cell diameter according to the resolution of the corresponding set. Instead, it was also necessary to adjust the threshold value for each set, so that the method could still detect the relevant cells. In this regard, ICS can be more generally applied than ITCN (see Table 3). 

## 3. Results

Table 1 shows a comparison between the counts returned by ITCN and ICS, including the processing times. ICS execution times depend directly on the resolution of the processed sample, and the software performs faster when the density of cells in the sample is high (14,900 cells and up). On the other hand, the execution times of ITCN depend more on the number of nuclei to be detected and to a lesser extent on the resolution of the input image. Consequently, when counting large number of cells in the range of tens of thousands, ITCN performs slower than ICS by a factor of three, and when counting small numbers of cells (1,700 or less), ITCN performs 2.6 to 3.6 times faster than ICS.

**Figure 8:**
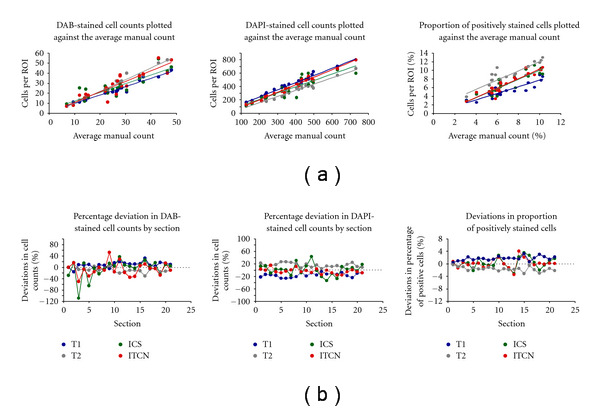
Comparison of automated methods to manual counts. (a) Regressions of the two automated counting methods (ICS and ITCN) and the individual manual cell counts (T1 and T2) against the average of the two manual counts by section. (b) Deviations from the average manual count for the two automated methods and the manual counts by section.

Figure 8 shows plots of cell counts and proliferation estimates obtained using the two automated methods as well as manual counts (T1 and T2). The overall impression from these plots is good concordance between the automated methods and mean manual counts.  Figure 8(b) shows the percentage deviation of the different counts from the average manual count. All count estimates show similar variation, but ICS shows a slightly higher variation than ICTN for the DAB and the DAPI count estimates.  Figure 10 (described below) explores this variation in more detail.  Table 2 shows the correlations between the mean manual counts and the counts from ICS and ITCN. The correlations between the automated methods and the mean manual counts for both DAB and DAPI stained cells are close to 0.9, while the cell proliferation estimates correlate less well at 0.7 for ICS and 0.8 for ITCN. 

**Figure 9:**
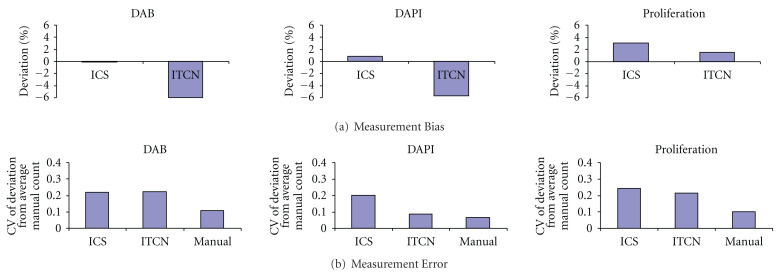
Measurement bias and error for automated and manual counts. (a) Measurement bias for automated counts using the ICS and ITCN methods. (b) Measurement error for ICS, ITCN, and manual cell counts.

**Figure 10:**
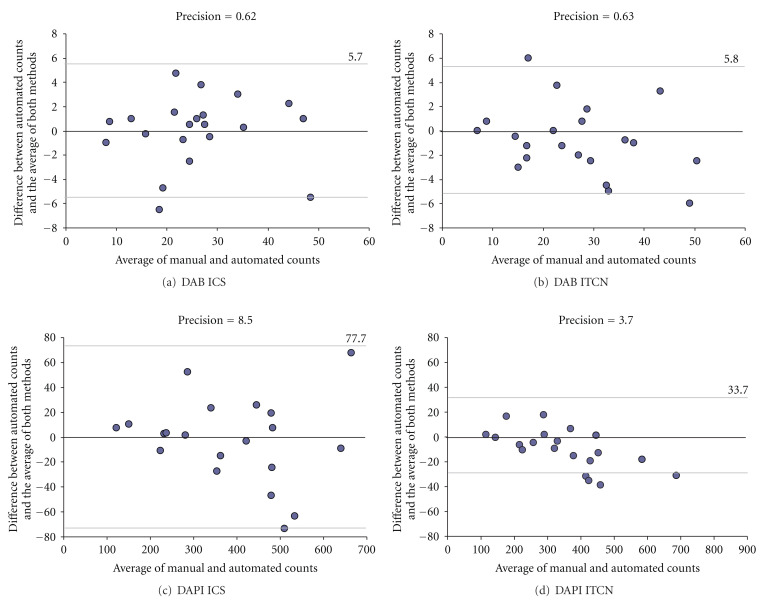
Bland-Altman plots of ICS and ITCN. **(**a) Difference between ICS counts and the average of all counts for DAB-stained samples. (b) Difference between ITCN counts and the average of all counts for DAB-stained samples. (c) Difference between ICS counts and the average of all counts for DAPI-stained samples. (d) Difference between ITCN counts and the average of all counts for DAPI-stained samples.

Figure 9 shows measurement bias for the two automated cell counts and both bias and error for both the automated and manual counts. Analysis of variance revealed that the bias differed significantly between the two methods for all three variables (*P* < .001). ICS is less biased overall than ITCN. However, the reason for the lower correlations of cell proliferation (Table 2) using ICS is that the small biases in the counts for ICS are in different directions for the DAPI- and DAB-stained cells, while the larger biases for ITCN are consistent underestimates for both image types. Levene's test revealed no significant difference in measurement error between the three methods for DAB (*P* = .286) and a marginally significant difference for DAPI (*P* = .045). Figure 10 shows the Bland-Altman plots of ICS and ITCN against the average of all counting methods. The results indicate similar precision (i.e., standard error) for both automated methods (0.62 for ICS and 0.63 for ITCN) when considering the DAB counts and a higher precision value for ICS (8.5) than for ITCN (3.7) when considering DAPI-stained samples.

Table 3 shows the results of running ICS and ITCN using the same set of 21 slice samples imaged under the two illumination conditions previously described.  Table 3 includes the results of manual counts (2 counts per observer, 2 observers) performed at 200x magnification. The average coefficient of variation (CV) for manual counts was 14.2% for DAB counts and 16.3% for DAPI counts. For the ICS counts of DAB-stained cells (brightfield microscopy) (26.7 cells per sample on average), the average CV was 8.9%. Average counts for the count of DAPI-stained nuclei (visible under UV light) were much higher (374 cells per sample), and ICS exhibited a CV of 2.7% (the lowest among all methods). The highest average CV values are observed for ITCN under both illumination conditions (19.3% for DAB and 10.9% for DAPI). 

## 4. Discussion

### 4.1. Accuracy and Precision of ICS Counts

The counts provided by ICS and the average of the counts made by manual counters were highly correlated (0.88). Overall, ICS produces less biased counts of cells using both imaging techniques than ITCN with comparable measurement error. This result suggests that ICS is more accurate than ITCN for estimating cell counts overall. However, due to the fact that the smaller biases present for ICS work in opposite directions for the two image types, the accuracy of the cell proliferation estimate is lower for ICS than ITCN. The precision of the cell count and cell proliferation estimates is comparable for both automated methods and for manual counting.  Table 3 shows that ICS provides the most consistent counts (observed as lower average coefficients of variation—CVs) across multiple resolutions, covering a wide range of cell diameters (from 6 to 32 pixels). When considering counts at multiple resolutions, ICS exhibits the lowest measurement error (average CV of 2.7%) when counting samples with hundreds of cells (samples of DAPI-stained tissue). For DAB counts at multiple resolutions, ICS also shows lower measurement error (8.9%) than manual counting (14.2%) and much lower measurement error than ITCN (19.3%). These statistical measurements, however, depend on the sample size. There is the possibility that the small differences actually found could become significant with a larger sample. With regard to the necessary precision for this study, there is no application-driven preference for false positives (over false negatives) or false negatives (over false positives). Both types of errors would be considered equally undesirable. However, our gold standard is the manual counting results, and since there is no significant difference in measurement error between the three counting methods (see Levene's test in Section 3), particularly between the manual counts and ICS counts, instances of false positives and false negatives seem to even out and are thus considered not relevant.

### 4.2. Advantages of ICS

**Figure 11:**
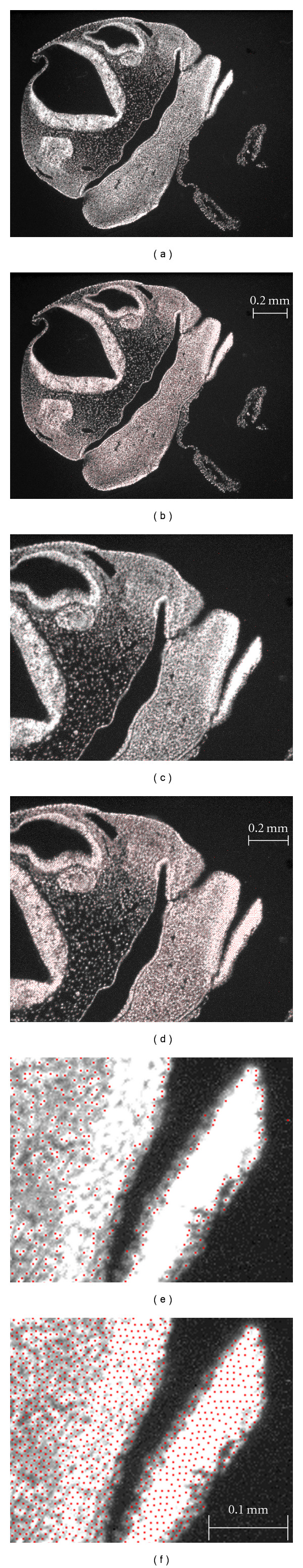
Estimates under saturated fluorescence. Comparison of cell counts estimates in regions with saturated fluorescence. Figures (a) and (b) show the original images produced by ITCN (a) and ICS (b). These images are of high resolution; thus, (c) and (e) show magnified views of ITCN, whereas (d) and (f) show magnified views from ICS. The red markers in (e) and (f) indicate cell locations identified by the ITCN and ICS, respectively. While ITCN only places markers on the borders of the saturated regions (e), ICS populates these regions by placing location markers according to the input cell diameter average (f).

For samples such as the one shown in Figure 3(b), most algorithms will have no problem counting cells which are clearly separated from the background and each other, while touching cells might be separated by using a distance transform and watershed algorithm. Other samples, such as the one shown in Figure 11, present a more challenging case for watershed algorithms due to the absence of information needed to separate the touching cells in the uniformly colored regions within the image. When viewing tissue sections at low magnifications under a UV source, it is not uncommon for fluorescent nuclei to appear to blend together such that their boundaries look fused with their neighbors. An advantage of ICS over edge detection-based methods such as ITCN is that ICS can place cell markers in parts of the image that are saturated with fluorescent signal (see comparison of Figures 11(e) versus 11(f)). Figure 11(f) shows a Poisson-disk (PD) distribution of cell markers in the saturated intensity (white) regions (ICS uses the average cell diameter to place markers for cell locations, so the radius of the PD distribution in these regions is half the average cell diameter). Conversely, ITCN can only detect nuclei located on the edges of these regions, but not within. Since ICS does not rely exclusively on the edge buffer information, it can place additional markers for cell locations within regions of saturated intensity based on the user-indicated cell diameter. This is a particular advantage of our algorithm over those that strongly depend on the detection of edges or a gradient vector field of the input image, for images that are somewhat saturated in some regions. However, further evaluation is needed to confirm this advantage over alternative methods and the accuracy of location identification.

Whereas ITCN can only be used to analyze images that have been converted to 8-bit grayscale (thereby losing color information), ICS can be used to count cells that have been stained to show different coloration under the same source. This means that the user can take a single image of a sample and obtain two or more cell counts, one for each of the two stains if both are visible on the slide. Further, the current implementation of ICS, also done in Java, can be executed from a command-line interface. This makes it possible to process large batches of images in parallel, provided they have uniform color intensities. Finally, in addition to producing an output image highlighting the detected cells, ICS provides a list of the cell locations in a convenient text file format. This information is used in the creation of a 3D point cloud of cell locations obtained by accumulating the data from a stack of sequential tissue samples. This facilitates further analysis of cell proliferation data in a way that not only considers the amount of cells in a sample, but also the location of these cells within a 3D volume, which is essential for morphometric analysis. 

### 4.3. Limitations of ICS

An inherent limitation of ICS is that it is appropriate for use only when the cells that are being analyzed can be characterized as having a certain average radius. Since cell types vary widely, the reader must double-check that this assumption can be made prior to using this method. 

ICS was tested for images produced at 200x magnification but has been designed to adapt to varying degrees of magnification. It is important to consider that when the intercellular distance is reduced to four pixels or less, which might happen at lower magnifications, the algorithm is bound to lose precision, as the traversal costs are computed on a per-pixel basis. To avoid this, we advise that the images given to ICS be taken at the highest resolution available in the microscope or scanner used. 

Since the color filter used by ICS is based on a user-provided color range, ICS depends on the application of a consistent illumination setting across samples. This can become a limitation if several samples are produced under inconsistent illumination settings or when illumination of the subject is inconsistent across the image. The images used in the study have some degree of variation in the intensity (see Figure 7), which are effectively dealt with by conservatively increasing the user-provided color range. We have applied our method to images with a higher degree of variation in the intensity distribution (see Figures 6 and 11). In some cases, intensity saturation or excess brightness in parts of the image can take place, as shown in Figure 11(f). The method deals effectively with these cases by placing estimated cell markers in saturated regions based on the average cell diameter. However, there is no protection for extremely low intensities within the sample; these regions would normally fall outside the average color range and would be undercounted. Such images would have to be subject to other algorithms aimed at correcting the intensity variation within the image prior to use with ICS. 

### 4.4. Future Work

As previously mentioned, an advantage of ICS is that it can be used to estimate cell counts in high-density regions of an input image where it is hard to assess the cell counts using standard edge detection-based algorithms. On the other hand, standard edge detection and segmentation-based algorithms (such as those in [3, 10–14]) have the advantage that they are better suited to deal with a higher degree of variation in the size and shape of the cells in the input images. Combining those two approaches through segmentation and separate processing of the input image might produce the best estimate of cell counts overall. Under this scheme, it could be possible to use ICS in a hybrid algorithm that relies heavily on ICS to process regions with low gradient intensities (low signal from the edge detection buffer) and edge-detection and segmentation approaches to process other regions of the images where cell density is smaller and the profiles of the cells are more clearly distinguishable.

In addition, ICS can complement texture analysis methods for cell counting (such as [16, 17]) by providing cell localization information. Under this scheme, texture analysis would be used to remove the background and segment the input image into regions encompassing different cell types. ICS would then populate the segmented regions according to the average size for cells in these regions. As mentioned earlier, such an approach would be relevant for developmental morphometric analysis.

## 5. Conclusions

We have developed Incremental Cell Search (ICS), a novel cell identification and counting algorithm used for histomorphometric analysis of embryonic tissue samples. ICS distributes markers in regions of interest that represent cells based on progressive algorithms used in image sampling and nonphotorealistic rendering. The regions of interest are defined by the user, who specifies the color range that identifies potential cells in the tissue and the estimated average cell diameter. The algorithm also contributes to finding cell location markers in regions with saturated fluorescence, although this feature has not been extensively validated. ICS use is appropriate when the cells or nuclei being identified are similar in size and can be approximated using round regions, as is the case in our application. Our results show that quick and consistent estimates of cell counts can be done using ICS on a variety of input images matching these criteria. 

##  Conflict of Interests

The authors declare that they have no conflict of interests.

##  Authors' Contributions

O. E. Meruvia-pastor, J. Soh, E. J. Schmidt, J. C. Boughner, M. Xiao, H. A. Jamniczky, B. Hallgrímsson and C. W. Sensen participated in the writing of the paper and in the design of the validation. E. J. Schmidt prepared the specimens for sampling, E. J. Schmidt and H. A. Jamniczky obtained the images, and E. J. Schmidt and J. C. Boughner performed the manual counts. O. E. Meruvia-Pastor wrote the method description and produced the counts using the automated software tools. B. Hallgrímsson and O. E. Meruvia-Pastor analyzed the results. B. Hallgrímsson and C. W. Sensen directed the research. The research was carried out at the University of Calgary, as well as most of the paper edition. All authors read and approved the final version of the paper.

## Figures and Tables

**Table 1:** Comparison between ITCN and ICS counts and processing times.

Image ID	Image size	ITCN		ICS	
		Number of nuclei	Processing time	Number of nuclei	Processing time
C-57-21	1600 × 1200	1,701	8 s	513	29 s
L-31	800 × 600	1,045	1.5 s	368	4 s
EDU	1280 × 1024	14,972	42 s	23,314	14 s

**Table 2:** Correlations for automated counts with the mean manual count and the ratio of proliferation. All are significant at *P* < .001.

Method	DAB counts	DAPI counts	Proliferation ratio
ICS	.878	.876	.705
ITCN	.902	.987	.776

**Table 3:** Count results of ICS and ITCN at different resolutions.

Resolutions			Manual counts		ITCN				ICS	
Cell diameter DAB (pixels)	Cell diameter DAPI (pixels)	Corresponding optical magnification	Brightfield (DAB stained)	UV (DAPI stained)	Brightfield (DAB stained)		UV (DAPI stained)		Brightfield (DAB stained)	UV (DAPI-stained)
					Threshold	Average count	Threshold	Average count	Average count	average count
45	32.4	400x	N/A	N/A	0.1	33.2	0	306.6	26.5	376.4
32	23	200x	26.0	411.5	0.2	33.6	0.1	261.0	26.2	375.3
25	16.2	100x	N/A	N/A	0.3	38.0	0.1	269.0	26.2	374.6
16	11.5	50x	N/A	N/A	0.8	36.7	0.1	288.7	27.7	376.6
8	5.75	12.5x	N/A	N/A	2	50.0	0.4	316.0	27.7	367.0

Average across resolutions:			26.0	411.5		38.3		288.3	26.8	374.0

Coefficient of variation across resolutions:			14.2%	16.3%		19.3%		10.9%	8.9%	2.7%
